# Vitamin D_3_, Vitamin K and Fatty Acid Profiles of *Nannochloropsis oceanica* Grown Under Outdoor Industrial Conditions

**DOI:** 10.3390/md24100349

**Published:** 2026-10-02

**Authors:** Marie Bagge Jensen, Moshe Meged, Dalia Malka, Na’ama Segal, Charlotte Jacobsen, Jette Jakobsen

**Affiliations:** 1 Research Group of Bioactives–Analysis and Application, Technical University of Denmark, 2800 Kongens Lyngby, Denmark; mabaj@food.dtu.dk (M.B.J.); chja@food.dtu.dk (C.J.); 2 Israel Oceanographic & Limnological Research Ltd.—National Center for Mariculture, Eilat 8811201, Israelsegaln@ocean.org.il (N.S.)

**Keywords:** raceway pond, menaquinone-4, long chain n-3 polyunsaturated fatty acids, UV, temperature, microalgae

## Abstract

*Nannochloropsis oceanica* is cultivated, at industrial scale, mainly for aquaculture feed and is known to contain different bioactive compounds, such as fat-soluble vitamins and long-chain polyunsaturated fatty acids. However, data on the variability and availability of these compounds over year-long outdoor cultivation conditions are absent. We conducted a longitudinal observational assessment of vitamin D_3_ and fatty acid content and composition of *N. oceanica* cultivated in an outdoor pilot scale pond in southern Israel (29.6° N). Cultivation cycles of 30 days were monitored across eight months between January and October. We further determined the vitamin K content during January and March. In freeze-dried biomass the vitamin D_3_ content ranged from 3 to 253 ng/gram and was positively associated with mean UV-index and pond pH. Menaquinone-4 was the only vitamin K vitamer detected (14–37 µg/gram) and was primarily associated with pond temperature. Total fatty acid in freeze-dried biomass was relatively constant at 7.6 ± 1.7% of which 1–3.5% was eicosapentaenoic acid (EPA). The fatty acid profile shifted during the 30-day cultivation period, with increasing relative proportions of EPA and decreasing proportion of saturated fatty acids. The results highlight substantial temporal and cultivation-related variability under real production conditions and provide a basis for controlled optimization studies.

## 1. Introduction

Over the past decade, the microalga *Nannochloropsis oceanica* (*N. oceanica*) has shown to be a promising source for valuable oil production targeting the biodiesel, nutraceutical, and aquaculture industries. This interest is driven by its notable capacity to accumulate lipids and a high percentage of polyunsaturated fatty acids (PUFAs), such as eicosapentaenoic acid (EPA) [1]. Furthermore, *N. oceanica* has been reported to synthesize several vitamins, including vitamin E and vitamin K, and can also produce vitamin D_3_ when exposed to ultraviolet light within the 280–315 nm range (UVB) [2,3,4,5].

Despite this potential, large-scale commercialization remains constrained by the time and cost associated with cultivation. *N. oceanica* utilises light, water, nutrients and CO_2_ to grow and synthesise bioactive compounds, and they therefore have the potential to serve as vegan cell factories for production of a range of the above-mentioned health promoting bioactive compounds [1].

Vitamin D and vitamin K are two groups of fat-soluble vitamers. Vitamin D plays an essential role for the homeostasis of calcium and phosphorus, and thereby healthy bone. Vitamin D can be produced in human skin by UVB-exposure. However, in Europe it is estimated that 40% of the population do not have a sufficient vitamin D status [6]. From the diet, the primary vitamin D vitamer, cholecalciferol (vitamin D_3_), is solely present in food of animal origin, while ergocalciferol (vitamin D_2_) is present in wild mushrooms, and in UVB-exposed cultivated mushrooms and yeast [7,8,9], but as mentioned *N. oceanica* could be a potential future vegan source.

Vitamin K is essential for blood coagulation and healthy bones and is associated with the prevention of cardiovascular diseases, cancer, and diabetes [10,11,12,13]. Vitamin K describes a group of vitamers that consists of phylloquinone (PK) and the menaquinones (MKs). Phylloquinone (PK) is abundant in green vegetables, whereas both PK and various menaquinones (MKs) are present in animal-derived foods and fermented products such as cheeses, as well as in microalgae [5,14,15,16,17,18]. Emerging evidence suggests that MKs may exert a more pronounced protective effect against the development of cardiovascular diseases [19,20], which has increased interest in identifying and promoting food sources rich in MKs.

Long chain n-3 PUFAs have been shown to reduce the risk of cardiac death, coronary heart disease, and ischemic stroke [21]. They have also been recognized to be important for mental health and are important for the development of vision and the brain of a foetus [22,23]. From the essential PUFA α-linolenic acid, humans can synthesize n-3 PUFAs such as EPA; however, this conversion is highly inefficient. Therefore, obtaining EPA directly through the diet is important, with particularly high levels found in fatty fish [24,25]. However, because overfishing poses a growing global threat [26], alternative sources of long-chain n-3 PUFAs are increasingly important to lessen the demand of fish and seafood. In this context, *N. oceanica* has emerged as a promising future source, and reports from investigation into the cultivation parameters have shown that by exposing *N. oceanica* to different abiotic stresses it is possible to alter their production of EPA [4,27]. Different facilities have been used to study these effects on the content of EPA, ranging from the use of a 1 L flask in a laboratory to a 950 L open pond [28,29,30,31,32].

In Israel, *N. oceanica* is cultivated at industrial scale to produce a protein source for aquaculture feed. Our findings for vitamin D, vitamin K and EPA under laboratory conditions [3,4,5] sparked an interest to investigate to what extent these bioactive compounds are formed in microalgae produced industrially in outdoor facilities.

To address this gap, we conducted a longitudinal observational study of an outdoor industrial *N. oceanica* cultivation system, monitoring seasonal and cultivation-related variation in lipid-soluble bioactive compounds under real production conditions. The Ultraviolet Index (UV index), which predicts the UV radiation levels on a 1–11+ scale [33,34], was used as an indicator of sunlight availability of the UVB relevant for photochemical conversion of its pre-cursor, 7-dehydrocholesterol, into vitamin D_3_ within the microalgae.

We investigated the effect of cultivation days and UV index over eight months, from January to October, on the content of vitamin D_3_ and fatty acid composition in biomass from outdoor-cultivated *N. oceanica.* In addition, vitamin K content was assessed during January and March. The results highlight substantial temporal and cultivation-related variability under real production conditions and provide a basis for future controlled optimization studies.

## 2. Results

### 2.1. Cultivation Conditions During Outdoor Production Cycles

**Figure A1:**
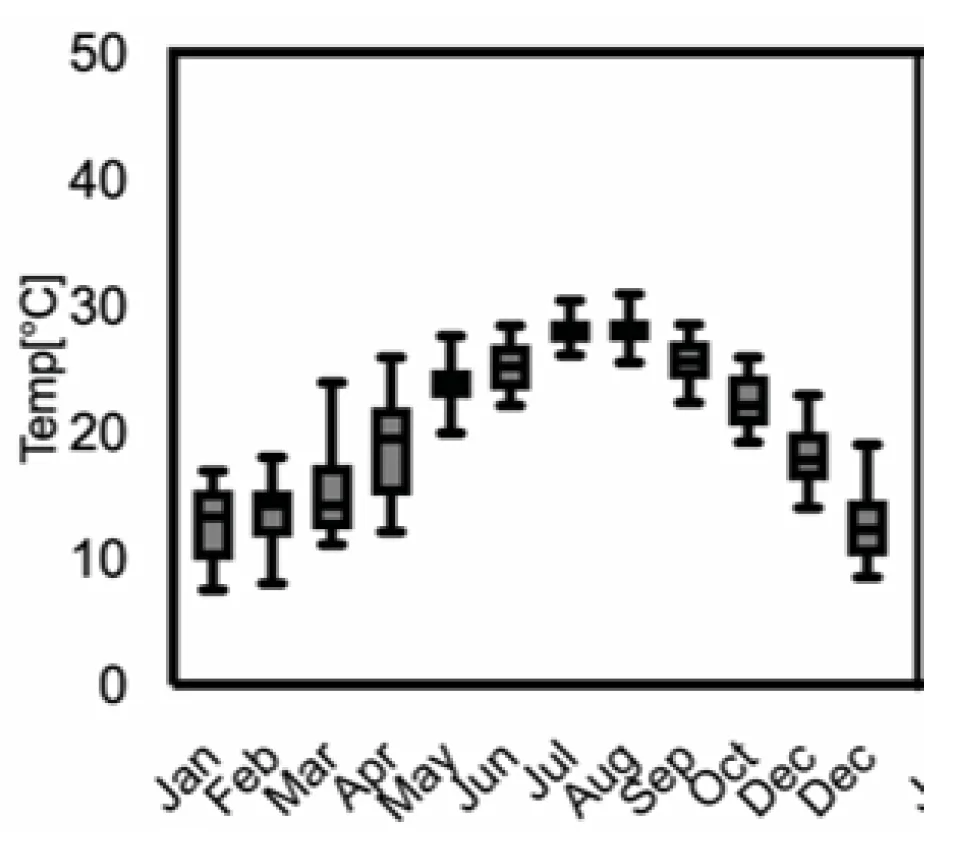
Ambient temperature during *Nannochloropsis oceanica* cultivation periods in Eilat, Israel, in 2021. Monthly variation in mean air temperature (minimum and maximum) recorded by the Israel Meteorological Service (IMS) meteorological station in Eilat during the study year.

Outdoor cultivation of *N. oceanica* was conducted in eight production cycles between January and October in 2021. Ambient temperatures are shown in Appendix A, Figure A1. Monthly mean air temperatures exhibited the expected seasonal variation, with lower values during winter months and higher values during summer.

During outdoor cultivation, monthly measurements were taken in the pond at the collection time points, including temperature, salinity, pH, and cell concentration. In addition, the mean UV index for the culture period up to harvest was estimated. Data are summarized in Table 1. Pond temperatures ranged from approximately 13 to 25 °C across the different months, reflecting seasonal changes in ambient conditions. Salinity, i.e., the concentration of sodium chloride (NaCl), ranges, with a mean from 36 to 52 g NaCl/L. The salinity remained within a relatively narrow range (±11%) within a month; a similar observation was seen for pond pH, which varied between monthly averages of 8.1 and 9.9, with only a minimal within-month variation (±7%). The average cell concentration ranged from approximately 48 × 10^6^ cells/mL to 107 × 10^6^ cells/mL per month but exhibited substantial within-month variability (±50%). In contrast, the estimated average UV index experienced by the culture ranged from 3.9 to 12 and showed only minimal variation within each month (±3%).

For cell concentration, the standard deviation exceeded the mean, reflecting high variability among observations. Because cell concentration cannot take negative values, the fact that the mean ± standard deviation interval extends below zero should not be interpreted as indicating negative concentrations but rather as reflecting a highly dispersed and potentially skewed distribution.

### 2.2. Vitamin D_3_ in Outdoor Cultivated N. oceanica

#### 2.2.1. Vitamin D_3_ Content During Cultivation Within and Between Months

**Figure 1:**
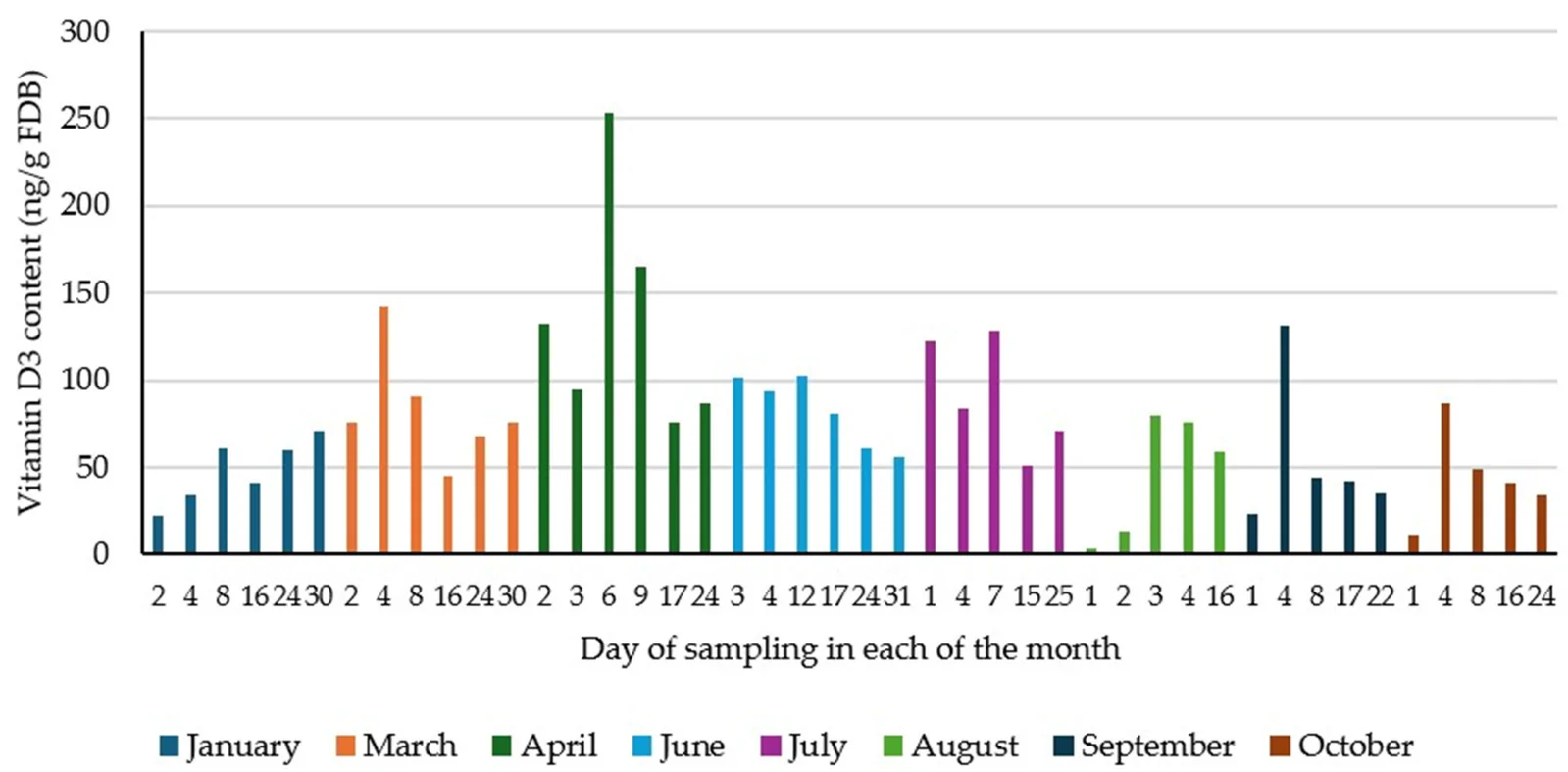
Vitamin D_3_ content measured in freeze-dried biomass (FDB) of *Nannochloropsis oceanica* in each of the samples collected from January to October.

To demonstrate the month-to-month variation in vitamin D_3_ content, the measured values for each of the eight months are shown in Figure 1.

The content of vitamin D_3_ ranged between 3 and 253 ng/g freeze-dried biomass of *N. oceanica* (FDB) representing the lowest in August (Day 1) and the highest in April (Day 6), while the maximum concentrations within each of the eight months ranged between 72 and 253 ng/g FDB in January and April, respectively (Figure 1). There is no association across the months between maximum content of vitamin D_3_ and mean UV index within a month (Pearson correlation 0.2; *p*-value 0.67).

#### 2.2.2. Effect of Cultivation Conditions on the Content of Vitamin D_3_ in *N. oceanica*

**Figure 2:**
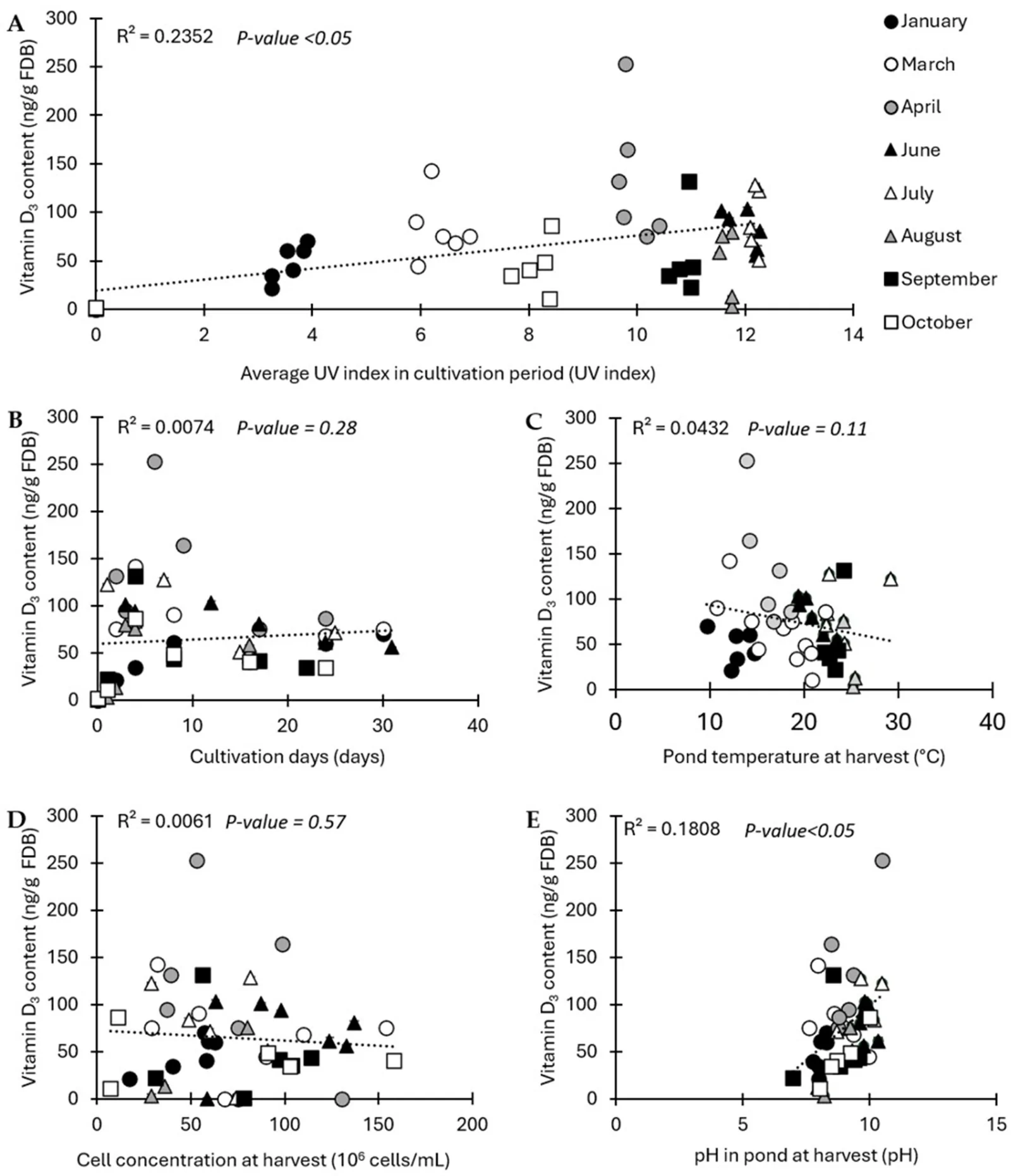
Factors affecting the vitamin D_3_ content of freeze-dried biomass of *N. oceanica* (FDB) cultivated outdoors from January to October. (**A**) The effect of average UV index in the cultivation period from day 1 until harvest; (**B**) the effect of cultivation days; (**C**) the effect of pond temperature at harvest; (**D**) the effect of cell concentration at harvest; and (**E**) the effect of pH in the pond. Linear regression is illustrated by the dashed line.

The relationships between the content of vitamin D_3_ in FDB and cultivation days, UV index, and temperature, cell concentration, and pH in the pond, respectively, are shown in Figure 2A–E. Samples from all eight months (January–October) were included in the test.

No effect on the vitamin D_3_ content in FDB was observed for the number of cultivation days (Figure 2B), pond temperature (Figure 2C), cell concentration in the pond (Figure 2D) nor salinity of the pond. In contrast a significant increase in the vitamin D_3_ content was observed when the average UV index during cultivation period increased (Figure 2A), and when the pH of the pond increased (Figure 2E).

### 2.3. Vitamin K in Outdoor Cultivated N. oceanica

Vitamin K was analysed in samples from January and March. MK-4 was present in *N. oceanica* while the contents of PK, MK-7 and MK-9 were below the limits of quantification, 1 ng/g, 5 ng/g and 5 ng/g, respectively.

Samples collected from the outdoor pond showed MK-4 levels of 23 µg/g and 21 µg/g FDB at Day 2 in January and March, respectively, and from Day 4 through Day 30, MK-4 concentrations ranged from 14 to 27 µg/g FDB. All results are available in Appendix A, Table A1.

**Figure A2:**
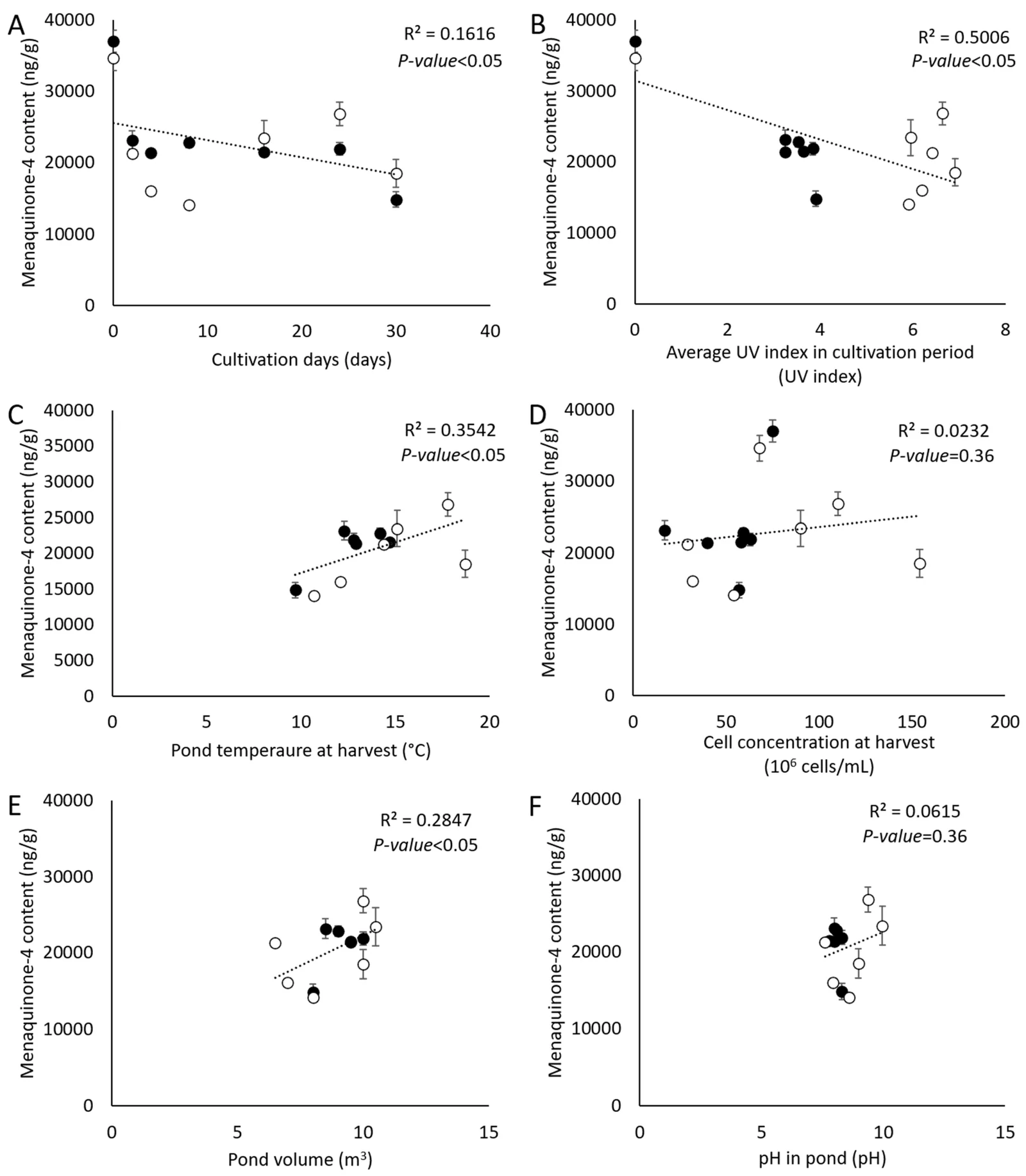
Factors effecting the menaquinones-4 (MK-4) content (ng/g) of freeze-dried *Nannochloropsis* *oceanica.* (**A**) The effect of number of cultivation days. (**B**) The effect of average UV index in the cultivation period. (**C**) The effect of pond temperature at harvest. (**D**) The effect of cell concentration at harvest. (**E**) The effect of pond volume. (**F**) The effect of the pH in pond. Error bars depict the range of the sample determinations (*n* = 2) for (**A**–**F**).

It was observed that pond temperature had significant effects on the content of MK-4 in *N. oceanica* (*p* < 0.05), while number of cultivation days (*p* = 0.86), average UV index (*p* = 0.47), cell concentration (*p* = 0.36), and pH (*p* = 0.36) showed no effect on the content of MK-4, as shown in Appendix A, Figure A2.

It is noteworthy that in both months, *N. oceanica* cultures had higher MK-4 levels at Day 0—before exposure to the outdoor light—measuring 37 µg MK-4/g in January and 35 µg MK-4/g in March. After two days in the outdoor pond (Day 2), these values had decreased to 23 µg MK-4/g and 21 µg MK-4/g, respectively.

### 2.4. Changes in Fatty Acid Content and Profile of N. oceanica During Cultivation

**Figure 3:**
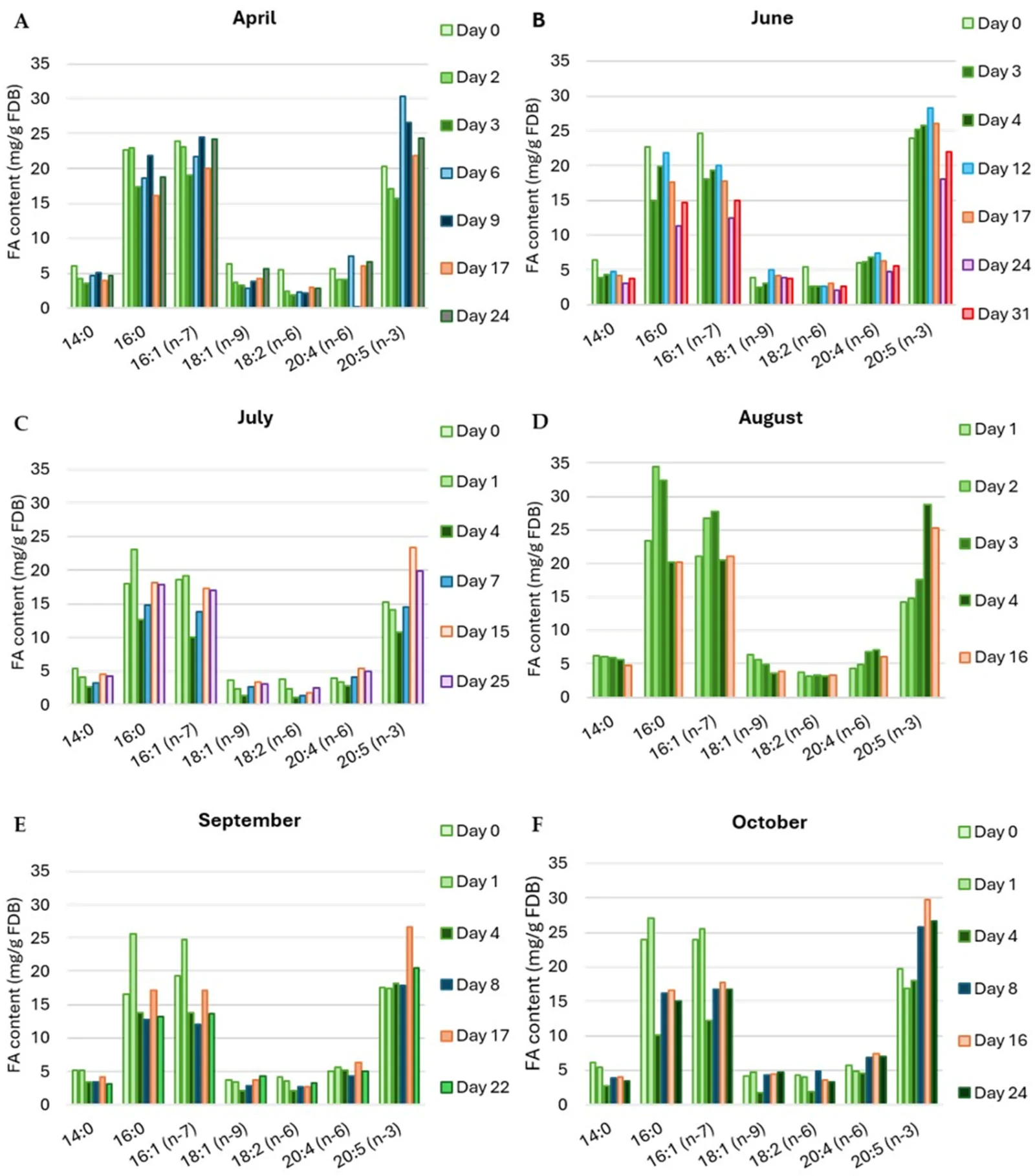
Content of fatty acid (FA) measured in the freeze-dried biomass of *Nannochloropsis oceanica* (FDB) cultivated outdoors over a cultivation period of up to 31 days. (**A**) In April; (**B**) in June; (**C**) in July; (**D**) in August; (**E**) in September; (**F**) in October.

The samples of *N. oceanica* collected in April, June, July, August, September, and October were analysed as FDB for 44 fatty acids. Seven fatty acids were quantified, comprising three PUFAs (EPA 20:5 (n-3)), arachidonic acid (20:4 (n-6)), and linoleic acid (18:2 (n-6)), two monounsaturated fatty acids (palmitoleic acid (16:1 (n-7)) and oleic acid (18:1 (n-9)), and two saturated fatty acids (myristic acid (14:0) and palmitic acid (C16:0)). Results are presented in Figure 3. The remaining 37 fatty acids were below the limit of quantification (0.055 mg/g).

In general, EPA content ranged from 10 to 35 mg/g FDB, while arachidonic acid and linoleic acid were present at 1 to 10 mg/g. Among the monounsaturated fatty acids, palmitoleic acid consistently exceeded oleic acid, and for the saturated fatty acids, palmitic acid was higher than myristic acid. Their concentrations fell within the same ranges of 10 to 35 mg/g FDB and 1 to 10 mg/g FDB, respectively.

It was observed that only the number of cultivation days had a significant effect on the content of EPA, myristic acid and palmitic acid. Across the different months a longer cultivation resulted in a significantly higher EPA content (*p* < 0.05) and significantly lower levels of the saturated fatty acids myristic acid and palmitic acid (*p* < 0.05). No significant effects were detected for arachidonic acid, palmitoleic acid, linoleic acid, or oleic acid (*p*-values 0.13–0.58).

The total fatty acid content was estimated at 7.6 ± 1.7 *w*/*w*% of FDB. This value was not influenced by the number of cultivation days, the average UV index during the cultivation period, or pond parameters such as pH, salinity, and cell concentration.

## 3. Discussion

### 3.1. Overview and Scope of the Study

The present study provides a longitudinal observational assessment of vitamin D_3_ and fatty acid composition and content in *N. oceanica* cultivated under outdoor industrial conditions and determines the vitamin K during two growth periods. Unlike controlled laboratory experiments, the study was conducted within the constraints of a commercial production system and was designed to capture temporal and seasonal variability rather than to establish causal relationships between individual environmental factors and metabolite production.

By monitoring multiple cultivation cycles across eight months and sampling repeatedly within each cycle for up to a 31-day production period, the study offers insight into how lipid-soluble bioactive compounds vary under realistic operational conditions.

### 3.2. Vitamin D_3_ Variability Under Outdoor Industrial Cultivation

Following UVB exposure and a temperature-dependent process, the precursor 7-dehydrocholesterol is converted to vitamin D_3_. It was therefore expected that the content of vitamin D_3_ in *N. oceanica* would increase with increasing UV index; however, we did not find any association between the mean ambient UV index in each month and the maximum vitamin D_3_ concentration observed within that month (Figure 1). In contrast, we did find the expected dependence for produced vitamin D_3_ on UV index collectively for the analysed samples collected from January through October (Figure 2A). The results were not as conclusive as those reported for laboratory scale UVB exposure of *N. oceanica* [3]. In the indoor experiment, the vitamin D_3_ content in *N. oceanica* increased from day 1 to day 5 when a constant UVB dose (15 kJ/m^2^/day) was applied and further increased when the UVB dose was increased to 36 kJ/m^2^/day.

As no other outdoor cultivation data are available for comparison with the maximum content of 253 ng vitamin D_3_/g FDB in our study, we compared our results with those reported from indoor cultivation studies. This comparison showed that the vitamin D_3_ achieved under outdoor condition was lower than obtained by indoor cultivation, where a final content of 1000 ng/g FDB was reached with a UVB dose of 36 kJ/m^2^/day when exposed for five days [3].

Importantly, the seasonal conditions in Eilat may explain the difference in the correlation between ambient UV index and vitamin D_3_ accumulation and experiments performed under controlled conditions. In spring (e.g., April), high irradiance coincides with moderate temperatures that promote rapid biomass growth, potentially increasing self-shading and reducing effective UVB exposure at the cellular level. Accordingly, ambient UV index alone may not accurately predict vitamin D_3_ production under outdoor conditions, and seasonal effects integrating both light and temperature should be considered. A significant positive association between pond pH and vitamin D_3_ content was observed, likely reflecting indirect coupling to photosynthetic activity rather than a direct effect of pH on vitamin D_3_ formation.

The statistically significant relationships indicate that UV-index and pH are associated with the variation in the vitamin D_3_ content. Nevertheless, the relatively low R^2^ values (0.23 and 0.18 for UV-index and pH, respectively) indicate that these variables explain only a limited proportion of the observed variability, suggesting that additional factors not included in the model are likely to play a substantial role in explaining the observed vitamin D_3_ content. It should be mentioned that there is a large difference between the amount of vitamin D produced by different microalgae species exposed to indoor UVB [5]. *N. oceanica* exposed to 15 kJ/m^2^/day for three days achieved a content of 285 ± 5 ng vitamin D_3_/g FDB, while *Nannochloropsis limnetica* achieved a content of 2700 ± 198 ng vitamin D_3_/g freeze-dried biomass and *Tetraselmis suecica* < 1 ng vitamin D_3_/g freeze-dried biomass [5]. The species selected for cultivation is therefore of great importance in relation to the production of vitamin D_3_.

### 3.3. Vitamin K (Menaquinone-4) Responses to Outdoor Cultivation

Menaquinone-4 was the only vitamin K vitamer detected in *N. oceanica* under the conditions examined, while phylloquinone and longer-chain menaquinones remained below the limit of quantification.

For the two months tested, in both cases a decrease in MK-4 content following the transfer from indoor to outdoor cultivation was observed, which is consistent with previous reports of reduced MK-4 concentrations under UV-exposed conditions [5]. This is presumably due to photodegradation, specifically the cleavage of the phytyl chain from the quinone moiety.

It is observed that the temperature of the pond significantly affected the content of MK-4 in *N. oceanica*, with a higher content of MK-4 achieved at higher temperature for the range from 8 °C to 19 °C (Appendix A, Table A1). Other studies have observed that suboptimal temperatures negatively affected the photosynthetic apparatus of *Monodus subterraneus* grown in outdoor ponds [36], and for *N. oceanica*, indoor cultivated the growth was depended on temperature in the range from 19 °C to 29 °C [37].

MK-4 and not PK has been shown to be the secondary electron acceptor in photosystem I in *Cyanidium caldarium* [38]. It might be that this is indeed the function of MK-4 in *N. oceanica*. Photosynthetic organisms acclimate to the conditions, such as light, temperature, and air, that they are exposed to.

### 3.4. Fatty Acid Content and Compositional Shifts During Outdoor Cultivation

The fatty acid profile of *N. oceanica* observed in this study was dominated by EPA, palmitic acid and palmitoleic acid, while docosahexaenoic acid (DHA), as expected, remained below the limit of quantification across all sampling points, which is consistent with previous reports for this species under both indoor and outdoor cultivation [39]. Absolute EPA contents ranged from approximately 1–3.5 *w*/*w*% of FDB.

During the outdoor cultivation period of up to 31 days, a consistent shift in fatty acid composition was observed, characterized by an increased relative contribution of polyunsaturated fatty acids, including EPA, and a corresponding decrease in the saturated fatty acids myristic and palmitic acid.

This temporal trend was evident across multiple cultivation cycles. In contrast, the total amount of fatty acid content remained stable, suggesting that outdoor cultivation primarily influenced fatty acid allocation rather than overall lipid accumulation. Others have shown that when decreasing the cultivation temperature or the light, the available content of EPA increases, but this study did not report on the effect on the content of saturated fatty acids [40].

Changes in fatty acid composition in microalgae are commonly associated with acclimation processes related to growth phase, light regime, nutrient availability and temperature. In the present study, cultivation time emerged as the dominant factor associated with compositional shifts, while no consistent associations were observed between fatty acid composition and individual environmental parameters such as mean ambient UV index, pond temperature or salinity. Given the observational nature of the study and the co-variation of environmental factors under industrial conditions, these findings should be interpreted as indicative of temporal acclimation rather than as evidence for specific causal drivers.

Previous laboratory studies have demonstrated that controlled manipulation of light intensity, spectral quality and nutrient status can significantly alter both lipid content and fatty acid composition in *Nannochloropsis* species [4,41]. The more moderate changes observed under outdoor industrial conditions in this study likely reflect the buffering effect of large culture volumes, self-shading and continuous mixing, which limit the extent of environmental stress experienced by individual cells.

From a production perspective, the observed stability of total fatty acid content, combined with a gradual increase in EPA proportion during cultivation, suggests that extending cultivation time may enhance EPA enrichment of biomass under outdoor conditions. However, further controlled experiments with defined replication and direct measurements of light and nutrient availability would be required to disentangle the relative contributions of growth phase and environmental drivers.

### 3.5. Limitations of the Present Study

The present study was conducted as an observational assessment of a single industrial outdoor cultivation system. Environmental variables co-varied seasonally and could not be independently controlled. In addition, independent biological replication at the pond level was not available at the site.

Due to limited resources, direct measurement of UV dose at the cultivation facility was not feasible. Instead, ambient meteorological data were used as proxies for outdoor conditions. As on-site UV measurement would have strengthened the analysis, such approaches should be incorporated in future studies to enable more accurate evaluation of the relationship between UV dose and biological response.

While the absence of independent pond-level replication limits statistical inference, the findings nonetheless provide valuable insight into variability under routine industrial cultivation conditions. They complement existing laboratory-scale studies by extending observations to a production-relevant context.

### 3.6. Is N. oceanica Produced in an Outdoor Industrial Raceway Nutritionally Relevant?

*N. oceanica* is well known to have a rigid cell wall, which limits the bioaccessibility of the nutrients within it [42]. While high-pressure homogenization enhanced the bioaccessibility of fatty acids from *Nannochloropsis* sp., the bioaccessibility achieved from digestion of the extracted oil was even higher [43]. This shows that the highest bioaccessibility of lipophilic compounds can be achieved when the lipophilic fraction is extracted from the microalgae and would therefore be a high value product that could be obtained from *N. oceanica*.

EFSA has set adequate intake levels of 15 µg vitamin D/day and 250 mg EPA plus DHA/day for adults. For vitamin K the recommended level is 70 µg/day [44].

The total content of fatty acids in the freeze-dried biomass of *N. oceanica* was in average 7.6 ± 1.7 *w*/*w*%. Using total fatty acids as an estimate for the lipid fraction of extracted oil from *N. oceanica* and hypothesizing that outdoor cultivation potentially could provide a stable high content of vitamin D_3_ one gram extracted oil would have a content up to 3.3 µg vitamin D_3_/g, up to 35 mg EPA/g, and up to 27 µg MK-4/g, thereby 4.5 g, 0.7 g and 0.2 g would contribute to approximately 100% of recommended dietary intake, respectively. Thus, five ml oil (one teaspoon) would be sufficient for all three nutrients. It is worth noting that the economic potential of such a product depends strongly on maintaining a stable and high nutrient content and achieving efficient oil extraction, among other factors.

Further processing and extraction of these compounds from *N. oceanica* may therefore be of great interest to be sold as a vegan alternative to other sources of these compounds.

## 4. Materials and Methods

### 4.1. Experimental Design

The present study was designed as a longitudinal observational assessment of an industrial *N. oceanica* cultivation system operated under commercial conditions. A single outdoor cultivation pond was monitored repeatedly across multiple production cycles lasting up to 31 days and conducted during eight months between January and October.

### 4.2. Cultivation System and Routine Production at the National Center for Mariculture

*N. oceanica* is routinely cultivated at the National Center for Mariculture (NCM), Eilat, Israel (29.6° N), as part of the aquaculture live-feed production chain, where it primarily serves as a feed source for rotifer cultures. The facility ranges from laboratory cultures (1 L) to industrial outdoor systems of up to 12,000 L.

Indoor cultivation is conducted in a dedicated growth room under controlled conditions, including constant illumination. A light intensity of 200 μmol photons/m^2^/s is measured in front of the carboys and Erlenmeyer flasks, while 150 μmol photons/m^2^/s is measured in front of the plastic-bags (HPL-225P, HOPTEK, Hangzhou Hopoo Light&Color Technology Co., Ltd., Hangzhou, China). Furthermore, the temperature is maintained at 25 °C, and the cultures are aerated with air enriched with approximately 3% CO_2_.

Cultures are typically scaled up through successive growth phases over a period of approximately 7 days until the target volume of 12,000 L required for outdoor transfer is reached.

Outdoor cultivation is conducted in raceway ponds (15 m × 4 m). Once cultures reach a minimum cell concentration of approximately 80 × 10^6^ cells mL^−1^, harvesting is initiated. Biomass is harvested using a Westfalia centrifuge (GEA Group, Boca Raton, FL, USA), with approximately 20% of the raceway volume removed per harvest event. Following harvesting, the raceway is replenished with freshly filtered seawater, and nutrients are added proportionally to the volume of water replaced to maintain continued growth. The ponds were enriched with CO_2_ for 10 min at the start of each hour during daylight hours. Pond volume varied within the 30-day cultivation period due to these scheduled additions of filtered seawater following partial harvesting events and was, on average, between 8500 L and 12,000 L within a single month. Throughout the cultivation period, pond volume was not routinely measured, and no water was added to compensate for evaporation-induced volume losses. The variation observed within each of the eight months is available in Appendix A, Table A2.

**Figure 4:**
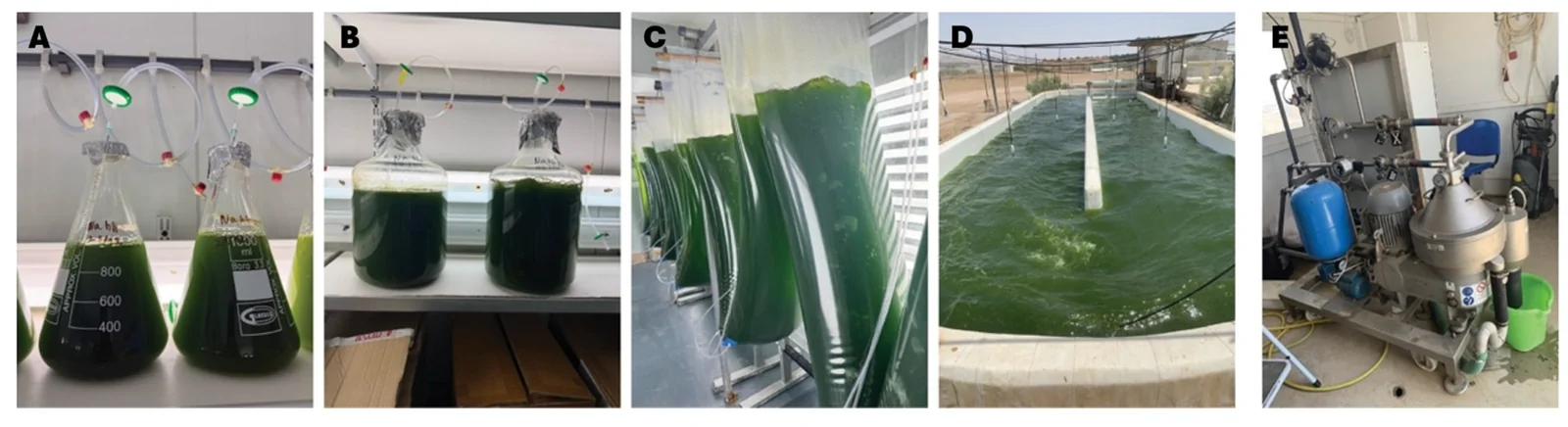
Overview of the *Nannochloropsis oceanica* cultivation system at the National Center for Mariculture, Eilat, Israel. (**A**–**C**) The indoor cultivation stages: (**A**) 1 L flask; (**B**) 5 L flask; and (**C**) 300 L plastic-bags. (**D**) The outdoor raceway pond used for large-scale production. (**E**) The Westfalia centrifuge employed for biomass harvesting.

This operational strategy reflects routine production conditions at the facility and allows for sustained biomass production under industrial outdoor cultivation, as described in Figure 4.

### 4.3. Measurement During Production

At harvest, the following cultivation conditions were measured in the outdoor pond: temperature, salinity of sodium chloride, using an Atago Salinity Refractometer S-10E (Atago, Tokyo, Japan), pH, and cell concentration, determined using a hemacytometer (Neubauer-Improved Counting Chamber, Marienfeld, Germany). In addition, the volume of the pond was noted before refilling with seawater.

### 4.4. Environmental Monitoring

Meteorological data for ambient air and temperature obtained for Eilat, Israel (29.5577° N, 34.9519° E) from the Israel Meteorological Service (ims.gov.il or weatheronline.co.uk) were used as external environmental descriptors rather than as direct measurements of in-pond conditions. For the statistical test, the average value was used (average UV index), which was calculated as the average of the extracted value for each day of the cultivation period.

### 4.5. Sampling

Sampling for vitamin and fatty acid analyses was collected from the indoor-grown culture prior to transfer to the outdoor raceway pond (day 0), and six additional sampling points during the outdoor cultivation period, planned for days 2, 4, 8, 16, 24, and 30.

Depending on pond conditions, the number of sampling points and sampling day may differ between each of the eight months.

### 4.6. Biomass Processing

During the indoor cultivation phase, culture volumes of approximately 20–30 L were harvested by batch centrifugation at 6000× *g* for 10 min in a Sorvall centrifuge (Sorvall, Thermo Scientific, Waltham, MA, USA) over several rounds. Larger indoor culture volumes (approximately 200–300 L) were processed using a continuous centrifuge at 12,000 rpm (Westfalia, GEA Westfalia Separator Group GmbH, Oelde, Germany). During the outdoor cultivation phase, samples of approximately 330 L were harvested using the outdoor continuous Westfalia centrifuge at 12,000 rpm.

Following continuous centrifugation, all samples were further concentrated by centrifugation at 6000× *g* (Sorvall, Thermo Scientific, Waltham, MA, USA) for 10 min. The resulting pellets were collected, freeze-dried (Alpha 1-2 LDplus, Martin Christ Gefriertrocknungsanlagen GmbH, Osterode am Harz, Germany), and shipped to the Technical University of Denmark (DTU). At DTU, they were stored at −20 °C until analysis within 12 months of collection.

### 4.7. Vitamin D Analysis

The vitamin D analysis was performed as previously described in references [45,46]. In short, 0.25 g of FDB was transferred to a 50 mL Sarstedt tube, followed by addition of 8 ng of the internal standard ^13^C_5_-vitamin D_3_ (-[23,24,25,26,27-^13^C_5_]-vitaminD_3_ (900234, Sigma-Aldrich, Burlington, MA, USA) and 0.2 g sodium ascorbate. The sample was then saponified at room temperature overnight. The vitamin D was extracted by a liquid–liquid extraction using ethyl acetate:*n*-heptane (20:80), cleaned by a HybridSPE-phospholipid column (Supelco, Bellefonte, PA, USA), and further derivatized with 4-phenyl-1,2,4-triazoline-3,5-dione (PTAD) for 5 min at room temperature in darkness. The vitamin D vitamers were then quantified by UHPLC-ESI-MS/MS (Agilent 1290 Infinity II UHPLC coupled to an Agilent 6470 triple quadrupole mass spectrometer; Agilent Technologies, Santa Clara, CA, USA).

The analyses for vitamin D_3_ were performed in random order in two batches; samples collected in January and March were analysed in triplicate, while samples collected from April through October were analysed in duplicate. In both cases, the analytical precision was <5%.

### 4.8. Vitamin K Analysis

The vitamin K analysis was performed as previously described in reference [15]. Briefly, 0.5 g FDB was weighed in a 15 mL Sarstedt tube followed by addition of 125 ng deuterium labelled (*d*7) PK, *d*7-MK-4, *d*7-MK-7, and *d*7-MK-9 as internal standards. The vitamin K vitamers were extracted by a liquid–liquid extraction using 2-propanol, *n*-heptane and water. The sample was then cleaned up by an SPE silica column (Biotage, Uppsala, Sweden), followed by UHPLC-ESI-MS/MS (Agilent 1290 Infinity II UHPLC coupled to an Agilent 6470 triple quadrupole mass spectrometer; Agilent Technologies, Santa Clara, CA, USA).

Analytical measurements were performed in triplicate and randomised for samples collected in January and March. The precision estimated as the pooled estimate for standard deviation at <5%.

### 4.9. Fatty Acid Analysis

The analysis of fatty acids was performed using direct transesterification of the lipids, as previously described in reference [47]. In brief, 0.045 g of FDB was weighed and dissolved in 1 mL methanolic sodium hydroxide (1 M). Next, 1 mL of toluene was added. A total of 2 µg of C23:0 was added as the internal standard, followed by ultrasonication, boiling at 100 °C for 2 min, cooling, and the addition of boron trifluoride for methylation. The fatty acid composition was analysed according to the principle of AOCS official method (Ce 1i-07) using GC-FID (HP-5890 A; Agilent Technologies, Santa Clara, CA, USA).

*N. oceanica* FDB collected in April, June, July, August, September and October was analysed in duplicate for 44 fatty acids, and the analyses were conducted in random order with a precision < 6%.

### 4.10. Statistical Analysis

Linear regression analysis was conducted to assess the effect (*p* < 0.05) of the different factors (number of cultivation days in each month, average UV index, and the pond parameters: temperature, cell concentration, salinity, and pH on the content of vitamin D for samples of *N. oceanica* collected January through to October, vitamin K for samples collected in January and March and fatty acids for samples collected April to October.

All statistical work was done using Excel (Microsoft^®^ Excel^®^ 2016).

## 5. Conclusions

Vitamin D_3_, MK-4, and EPA were found in freeze-dried biomass of *N. oceanica* that had been cultivated outdoors in Israel. It was further shown that the content of vitamin D_3_ in *N. oceanica* grown outdoors in Israel was dependent on the number of cultivation days and the average UV index during the cultivation period. The amount of MK-4 in *N. oceanica* grown in outdoor facilities in Israel was independent of parameters like UV index, cultivation days, and the pond temperature, salinity and pH. The total fatty acid content did not differ in the *N. oceanica* cultivated at different times of the year. However, the profile of fatty acid was dependent on cultivation duration, showing an increase in omega-3 fatty acid content (EPA) and a concurrent decrease in the saturated fatty acids myristic and palmitic acid throughout the up to 31-day cultivation period.

The total fatty-acid content remained stable throughout the year, averaging 7.6 ± 1.7% of the freeze-dried biomass.

The results indicate that one teaspoon of oil from freeze-dried biomass of *N. oceanica* could provide the recommended dietary intake for vitamin D, K as well as EPA. Achieving such levels requires an extraction process that involves breakdown of the microalgae cell wall for the oil to become a vegan source of lipid soluble health promoting nutrients.

## Figures and Tables

**Table 1:** For each month of *Nannochloropsis oceanica* grown in an outdoor pond. The table presents the number of samplings and sampling period and the mean and standard deviation of the temperature, salinity, pH, harvest cell concentration, and estimated UV index.

	January	March	April	June	July	August	September	October
**Number of sampling points ^1^**	6	6	6	6	5	5	5	5
**Sampling period, day**	2–30	2–30	2–24	3–31	1–25	1–16	1–24	1–22
**Temperature, °C**	13 ± 1.8	15 ± 3.1	16 ± 1.8	21 ± 1.6	24 ± 2.9	25 ± 0.8	23 ± 0.8	21 ± 1.1
**Salinity, g NaCl/L**	45 ± 0.8	52 ± 3.3	52 ± 3.9	41 ± 9.7	40 ± 4.0	41 ± 4.8	38 ± 4.4	36 ± 3.4
**pH**	8.1 ± 0.2	8.7 ± 0.9	9.2 ± 0.7	9.9 ± 0.2	9.7 ± 0.7	8.4 ± 0.6	8.8 ± 0.9	8.9 ± 0.7
**Harvest cell concentration, 10^6^ cells/mL**	49 ± 18	78 ± 63	61 ± 43	107 ± 27	62 ± 40	48 ± 57	80 ± 44	74 ± 87
**UV index ^2^**	3.9 ± 0.4	6.9 ± 1.1	10 ± 0.7	12 ± 0.5	12 ± 0.4	12 ± 0.4	11 ± 0.5	7.7 ± 0.6

^1^ Number of sampling points from the outdoor pond. ^2^ Mean value during each cultivation period, estimated from environmental data for Eilat, Israel [35].

## Data Availability

The raw data supporting the conclusions of this article will be made available by the authors on request.

## Abbreviations

The following abbreviations are used in this manuscript:

FDB	Freeze-dried biomass of *Nannochloropsis oceanica*
PUFA	Poly unsaturated fatty acids
EPA	Eicosapentaenoic acid
PK	Phylloquinone
MK	Menaquinone

## Appendix A

**Table A1:** Content of menaquinone 4 in freeze-dried *Nannochloropsis oceanica* collected in January and March.

Month	Cultivation Day	ng MK-4/g FDB ^1^	% sd	Temperature, °C
January	0	37,001	4	Indoor
January	2	23,153	6	12.3
January	4	21,362	1	12.9
January	8	22,807	3	14.2
January	16	21,511	1	14.7
January	24	21,897	4	12.8
January	30	14,360	4	9.7
March	0	34,623	5	Indoor
March	2	21,246	3	14.4
March	4	16,023	1	12.1
March	8	14,099	4	10.7
March	16	21,319	4	15.1
March	24	26,830	6	17.8
March	30	18,525	10	18.7

^1^ FDB: Freeze-dried biomass of *Nannochloropsis oceanica*.

**Table A2:** Pond volume (mean ± sd) at sampling during the cultivation January through October 2021.

Month	Pond Volume, m^3^
January	9.1 ± 0.7
March	8.7 ± 1.7
April	10 ± 1.1
June	8.5 ± 1.2
July	9.9 ± 1.3
August	8.9 ± 1.9
September	11 ± 2.3
October	11 ± 2.2
